# Anesthesia-Related and Perioperative Cardiac Arrest in Low- and High-Income Countries: A Systematic Review With Meta-Regression and Proportional Meta-Analysis

**DOI:** 10.1097/MD.0000000000001465

**Published:** 2015-09-11

**Authors:** Fernando A. Koga, Regina El Dib, William Wakasugui, Cairo T. Roça, José E. Corrente, Mariana G. Braz, José R.C. Braz, Leandro G. Braz

**Affiliations:** From the Anesthesia Cardiac Arrest and Mortality Study Commission (FAK, RED, WW, CTR, MGB, JRCB, LGB), Department of Anesthesiology, Botucatu Medical School, Univ Estadual Paulista (UNESP); and Department of Biostatistics (JEC), Institute of Biosciences, Univ Estadual Paulista (UNESP), Botucatu, Brazil.

## Abstract

The anesthesia-related cardiac arrest (CA) rate is a quality indicator to improve patient safety in the perioperative period. A systematic review with meta-analysis of the worldwide literature related to anesthesia-related CA rate has not yet been performed.

This study aimed to analyze global data on anesthesia-related and perioperative CA rates according to country's Human Development Index (HDI) and by time. In addition, we compared the anesthesia-related and perioperative CA rates in low- and high-income countries in 2 time periods.

A systematic review was performed using electronic databases to identify studies in which patients underwent anesthesia with anesthesia-related and/or perioperative CA rates. Meta-regression and proportional meta-analysis were performed with 95% confidence intervals (CIs) to evaluate global data on anesthesia-related and perioperative CA rates according to country's HDI and by time, and to compare the anesthesia-related and perioperative CA rates by country's HDI status (low HDI *vs* high HDI) and by time period (pre-1990s *vs* 1990s–2010s), respectively.

Fifty-three studies from 21 countries assessing 11.9 million anesthetic administrations were included. Meta-regression showed that anesthesia-related (slope: −3.5729; 95% CI: −6.6306 to −0.5152; *P* = 0.024) and perioperative (slope: −2.4071; 95% CI: −4.0482 to −0.7659; *P* = 0.005) CA rates decreased with increasing HDI, but not with time. Meta-analysis showed per 10,000 anesthetics that anesthesia-related and perioperative CA rates declined in high HDI (2.3 [95% CI: 1.2–3.7] before the 1990s to 0.7 [95% CI: 0.5–1.0] in the 1990s–2010s, *P* < 0.001; and 8.1 [95% CI: 5.1–11.9] before the 1990s to 6.2 [95% CI: 5.1–7.4] in the 1990s–2010s, *P* < 0.001, respectively). In low-HDI countries, anesthesia-related CA rates did not alter significantly (9.2 [95% CI: 2.0–21.7] before the 1990s to 4.5 [95% CI: 2.4–7.2] in the 1990s–2010s, *P* = 0.14), whereas perioperative CA rates increased significantly (16.4 [95% CI: 1.5–47.1] before the 1990s to 19.9 [95% CI: 10.9–31.7] in the 1990s–2010s, *P* = 0.03).

Both anesthesia-related and perioperative CA rates decrease with increasing HDI but not with time. There is a clear and consistent reduction in anesthesia-related and perioperative CA rates in high-HDI countries, but an increase in perioperative CA rates without significant alteration in the anesthesia-related CA rates in low-HDI countries comparing the 2 time periods.

## Abstract 2

Supplemental Digital Content is available in the text

## INTRODUCTION

The global anesthesia-related cardiac arrest (CA) rate may be a quality indicator to improve patient safety in the perioperative period. Considering the differences in surgery and anesthesia safety among countries,^1,2^ the country development status was assigned according to the Human Development Index (HDI) set by the United Nations Development Programme.^3^ This is a reliable worldwide marker of a national development status index based on per capita income, literacy, life expectancy, and enrollment in further education. HDI scores range from 0 to 1 (with 0 and 1 representing, respectively, the lowest and highest levels of development), and the countries with HDI ≥0.8 are considered to present very high human development.^3^ Therefore, as previously described,^4^ high-income countries were defined as HDI ≥0.8 and low-income countries as HDI <0.8. Some individual studies from a high-income country (the United States) report that anesthesia-related and perioperative CA rates have declined over time.^5,6^ A recent systematic review and meta-analysis of the literature has revealed a reduction in the risk of perioperative and anesthesia-related mortality over the past 50 years, with the greatest decline in high-income countries.^4^ However, a systematic review with meta-analysis of the worldwide literature related to global anesthesia-related CA rate has not yet been performed. We tested the hypothesis that anesthesia-related and perioperative CA rates decrease in high- and low-income countries over time by conducting a meta-analysis and meta-regression of cohort and cross-sectional studies.

The aim of this study was to analyze, by meta-regression, global data on anesthesia-related and perioperative CA rates according to country's HDI and by time, and also to compare the anesthesia-related and perioperative CA rates in low- and high-income countries according to country's HDI status in 2 time periods (pre-1990s *vs* 1990s–2010s), through proportional meta-analysis.

## METHODS

The reporting of present review adhered to the preferred reporting items for systematic reviews and Meta-Analyses of Observational Studies in Epidemiology statements.

### Ethics

Ethical approval was not necessary because this is a systematic review.

### Literature Search and Selection Criteria

Using medical subject heading terms and text words, including an exhaustive list of synonyms (Supplemental Digital Content 1, http://links.lww.com/MD/A397), we performed a systematic search to identify all studies that reported anesthesia-related and perioperative CA rates. The search strategy was adapted to each database to find related articles.

Two investigators (FAK and LGB) searched the US National Library of Medicine (MEDLINE, from 1966 to October 2014), Excerpta Medica Database (EMBASE, from 1974 to October 2014), Scientific Electronic Library Online (SCIELO, from 1997 to October 2014), and the Latin American and Caribbean Health Sciences Literature Database (*Literatura Latino-Americana e do Caribe em Ciências de Saúde*—LILACS, from 1982 to October 2014). The date of the last search was October 2, 2014. In addition, we manually reviewed the references of each article and included the related articles. There were no restrictions on either language or year of publication. When necessary, we used translation services at our institution.

Studies were included if they fulfilled the following a priori criteria: cohort and cross-sectional studies that reported anesthesia for surgery in a hospital setting; studies that specified anesthesia-related and/or perioperative CA rates until the seventh postoperative day; and studies with sufficient information to calculate these data.

Studies were excluded if they met any of the following criteria: focused on specific age groups (eg, children only); reported only 1 surgical procedure (eg, cardiac surgery) or a specific anesthetic technique (eg, regional anesthesia) or patient subtype (eg, a patient with American Society of Anesthesiologists [ASA] physical status I and II only); studies without the time period specified; or studies evaluating <3000 patients. The minimum sample size of 3000 patients for each study was chosen to estimate a rare adverse event (≤1 per 1000 anesthetics) in accordance with the rule of 3 sample size approximations.^7^

### Data Extraction and Outcome Definitions

Two authors independently searched and retrieved references to identify included studies. They used standard forms to extract information to identify the author(s), recruitment year(s), publication year, country of origin, and data source. Disagreements between the 2 authors were resolved by discussion, and a consensus was reached in all cases.

The primary outcome was anesthesia-related CA defined, according to the authors of the studies included in the review, as an event attributable to anesthesia that can be total or contributory. The former is defined as CA attributable only to anesthesia, whereas the latter is defined as CA partially attributable to anesthesia. The secondary outcome was perioperative CA, which was defined as CA from any cause (patient disease/condition, surgery, and anesthesia).

High-income countries were defined as HDI ≥0.8 and low-income countries as HDI <0.8. As country's HDI can change over time and many studies reported data over a time period of several years, the HDI for each study was assigned as the mean of the HDI values between the first and last year in which the patients were recruited. If the HDI values were not available for the specific time period of the study, the HDI from the closest date available was used.

The time frame for the achievement of the results of this study was dichotomized into 2 time periods (pre-1990s *vs* 1990s–2010s) as a variety of safety improvement measures that have emerged from the early 1990s in high-income countries and after a while in some low-income countries. This involved the organization of services for the care of patients, including the operating room (OR) materials and equipments such as anesthesia workstations with ventilators to provide adequate ventilation and monitoring, new drugs for anesthesia induction, and increasing the number of adult and child intensive care beds.^8–10^

### Statistical Analysis

We performed a meta-regression with a fixed-effect model using restricted estimated maximum likelihood with an observed log-odds ratio to predict whether CA rates changed significantly by time or country's HDI status (time and HDI as continuous variables). Meta-regression analysis was performed using Stata-13 (StataCorp LP, College Station, TX).

In addition, we used a random-effects model to calculate weighted event rates across all studies to perform a proportional meta-analysis using the pooled analysis of proportions.^11–14^ The time and HDI were dichotomized (pre-1990s *vs* 1990s–2010s and low HDI *vs* high HDI, respectively) to further evaluate anesthesia-related and perioperative CA rates. The event rate was defined as the number of CA per 10,000 anesthetics; data were reported with their corresponding 95% confidence intervals (CIs). The differences in proportions were compared using the χ^2^ test to compare the events for each time period or country's HDI. When data were provided only in aggregate time intervals (eg, from June 6, 1987 to June 6, 1991), the data were assigned to the median year of the study's patient recruitment interval (ie, median: 1989).^4^

An alternative approach that quantifies the effect of heterogeneity is called *I*^2^, which indicates the proportion of variability between studies resulting from heterogeneity rather than sampling errors.^15,16^*I*^2^ values >50% suggest significant heterogeneity among studies.

StatsDirect (StatsDirect Ltd, Altrincham, Cheshire, UK) was used to plot the studies into a proportional meta-analysis. The proportion tests were performed using Statistical Analysis System (SAS) for Windows®, v.9.2 (SAS Institute, Cary, NC). Statistical significance was defined as *P* < 0.05.

## RESULTS

**FIGURE 1:**
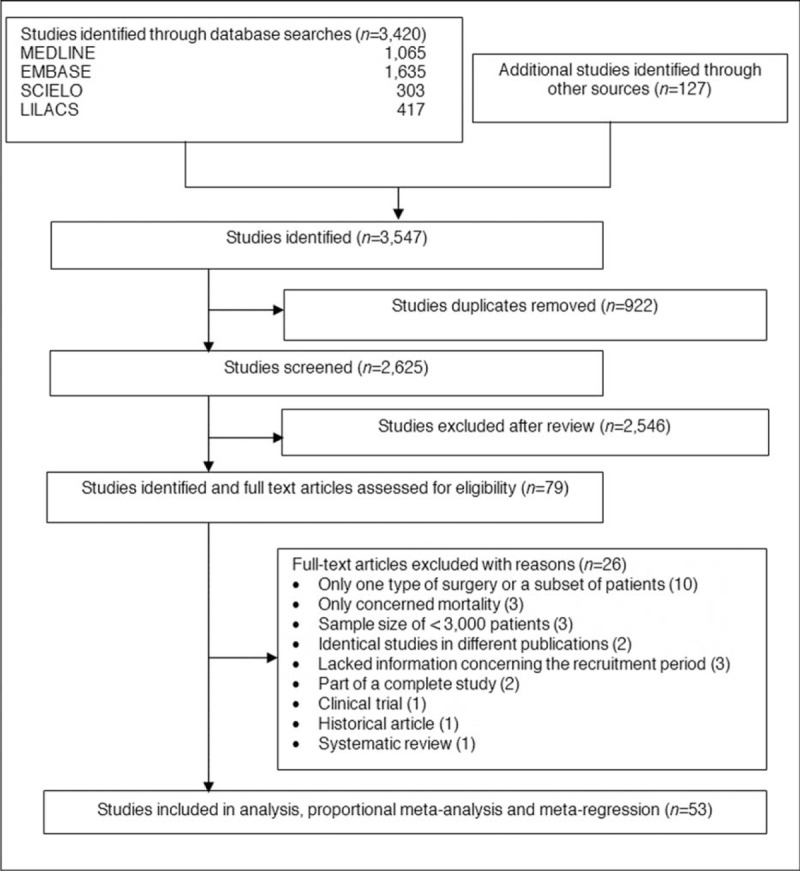
Flowchart of study identification.

Our search strategy yielded 3547 citations. We retrieved 79 publications for detailed evaluation (Figure 1). Of these articles, 53 studies from 21 countries met the inclusion criteria. In these 53 studies, 11,975,964 anesthetics were administered to patients who underwent anesthesia for surgery. The forest plot charts are presented in the Supplemental Digital Content 2, http://links.lww.com/MD/A397, to summarize the data and in the Supplemental Digital Content 3, http://links.lww.com/MD/A397, listed the characteristics and designs of these studies, with the earliest study being published in 1952 and the most recent in 2014. As expected, the proportion because of heterogeneity *I*^2^ presented a minimum of 81.5% and a maximum of 99.0% for all event rates (Table 1).

**TABLE 1:**
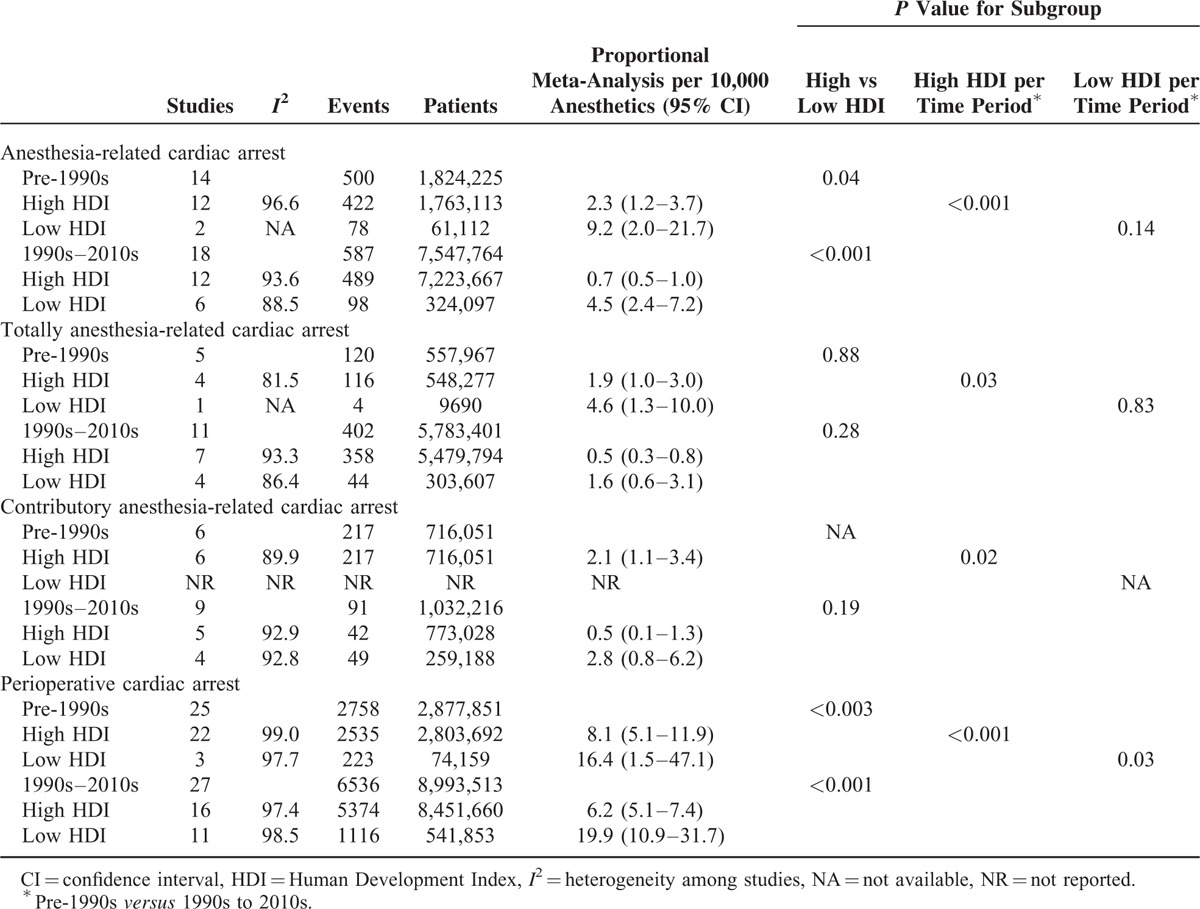
Proportional Meta-Analysis of Anesthesia-Related and Perioperative Cardiac Arrests by Time Period and by Country's Human Development Index Status

### Meta-Regression Analysis

#### HDI Status

**FIGURE 2:**
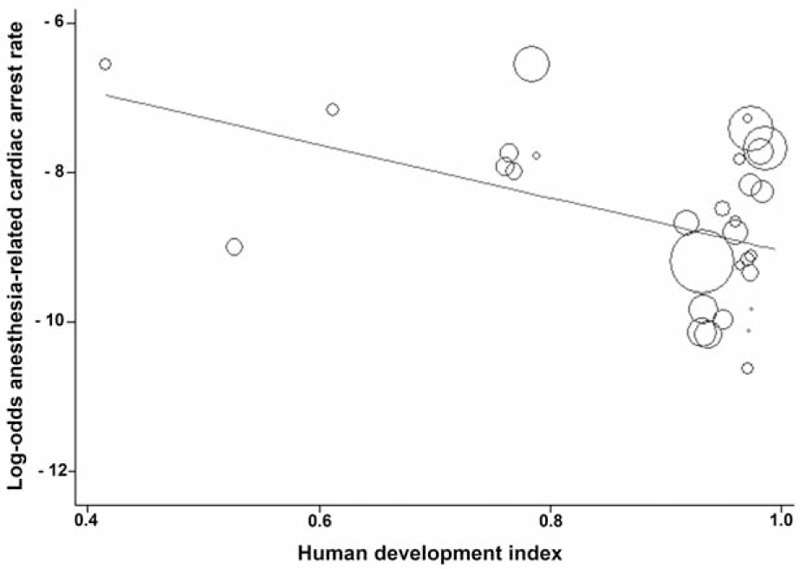
Meta-regression of anesthesia-related cardiac arrest rate by country's Human Development Index status. Each circle represents a study highlighted by its weight in the analysis. The relationship between anesthesia-related cardiac arrest rate and Human Development Index was significant (slope: −3.5729; 95% confidence interval: −6.6306 to −0.5152; *P* = 0.024).

**FIGURE 3:**
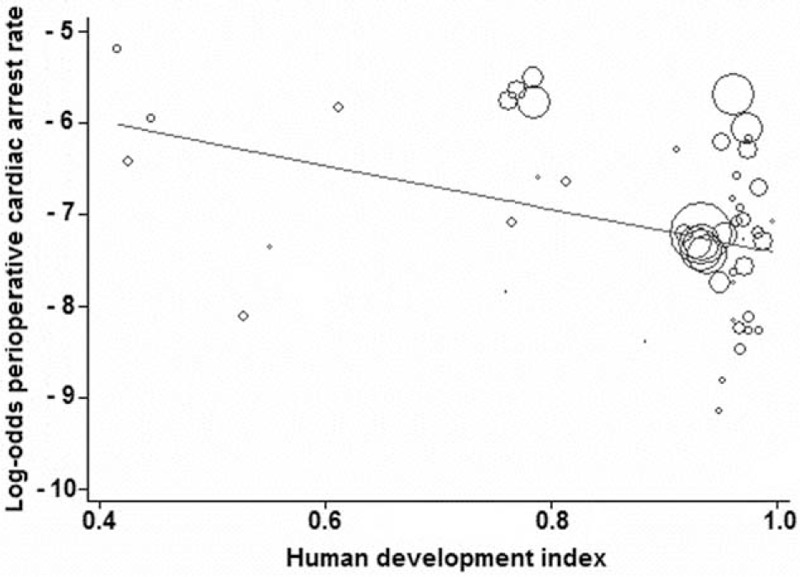
Meta-regression of perioperative cardiac arrest rate by country's Human Development Index status. Each circle represents a study highlighted by its weight in the analysis. The relationship between perioperative cardiac arrest rate and Human Development Index was significant (slope: −2.4071, 95% confidence interval: −4.0482 to −0.7659; *P* = 0.005).

Data from studies assessed in a weighted meta-regression showed that the relationship between anesthesia-related CA rate decreased significantly with increasing HDI (slope: −3.5729; 95% CI: −6.6306 to −0.5152; *P* = 0.024; Figure 2), whereas the relationship between totally anesthesia-related CA rate and HDI was not significant (slope: −1.2164, 95% CI: −5.5644 to 3.1316; *P* = 0.558). Similarly, the relationship between the contributory anesthesia-related CA rate and HDI was not significant (slope: −0.7399, 95% CI: −5.3161 to 3.8362; *P* = 0.729). However, perioperative CA rate showed a significant reduction as the HDI increased (slope: −2.4071, 95% CI: −4.0482 to −0.7659; *P* = 0.005; Figure 3).

#### Time

**FIGURE 4:**
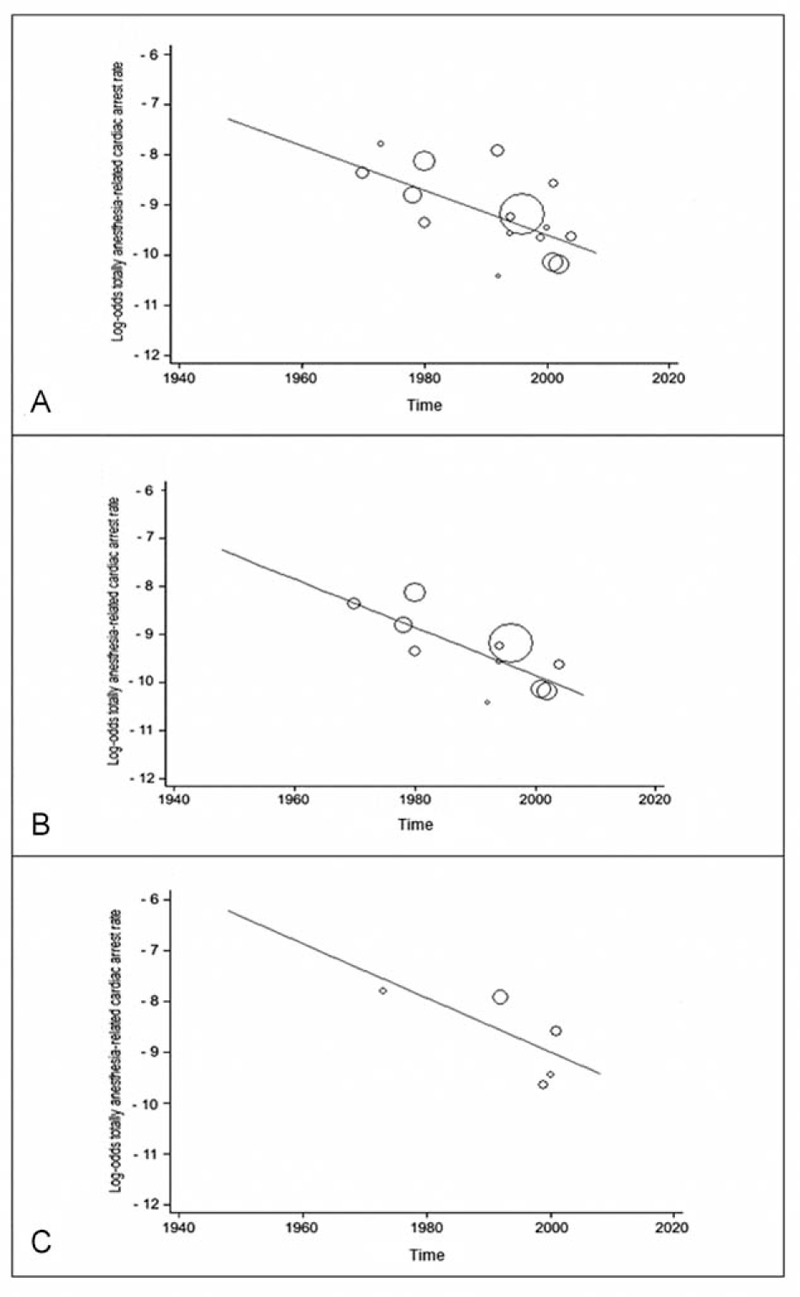
Meta-regression of totally anesthesia-related cardiac arrest rate by study year. Each circle represents a study highlighted by its weight in the analysis. (A) The relationship in all included studies regardless the Human Development Index status was significant (slope: −0.0446; 95% confidence interval: −0.0816 to −0.0075; *P* = 0.021); (B) the relationship in high-Human Development Index studies was significant (slope: −0.0501; 95% confidence interval: −0.0852 to −0.0149; *P* = 0.01); (C) the relationship in low-Human Development Index studies was not significant (slope: −0.0533; 95% confidence interval: −0.1579 to 0.0512; *P* = 0.203).

Data from studies assessed in a weighted meta-regression showed that the relationship between anesthesia-related CA rate and time was not significant (slope: −0.0098, 95% CI: −0.0449 to 0.0252; *P* = 0.304). However, there was a significant reduction of totally anesthesia-related CA rate by time independently of HDI status (slope: −0.0446; 95% CI: −0.0816 to −0.0075; *P* = 0.021), as well as high-HDI country rates (slope: −0.0501; 95% CI: −0.0852 to −0.0149; *P* = 0.01), but not with the low-HDI country rates (slope: −0.0533; 95% CI: −0.1579 to 0.0512; *P* = 0.203; Figure 4). The relationship between the contributory anesthesia-related CA rate and time (slope: −0.0058, 95% CI: −0.0485 to 0.0369; *P* = 0.770) was not significant. Similarly, perioperative CA rate by time was not significant (slope: 0.0089, 95% CI: −0.0083 to 0.0262; *P* = 0.304).

### Proportional Meta-Analysis (Table 1)

When comparing the studies by country's HDI status per time period (pre-1990s *vs* 1990s–2010s), the proportional meta-analysis showed that anesthesia-related CA rates decreased 3.2-fold in high-HDI countries (2.3 per 10,000 anesthetics before the 1990s to 0.7 per 10,000 anesthetics in the 1990s–2010s; *P* < 0.001), but only 2-fold in low-HDI countries (9.2 per 10,000 anesthetics before the 1990s to 4.5 per 10,000 anesthetics in the 1990s–2010s), which was not significant (*P* = 0.14) (Table 1). The event rates were 4-fold higher before the 1990s (*P* = 0.04) and 6.4-fold higher from the 1990s to 2010s (*P* *<* 0.001) in the low-HDI countries when compared with high-HDI ones.

Totally anesthesia-related CA rates decreased 3.8-fold in the high-HDI countries (1.9 per 10,000 anesthetics before the 1990s to 0.5 per 10,000 anesthetics in the 1990s–2010s; *P* = 0.03), but not significantly in the low-HDI countries ones (4.6 per 10,000 anesthetics before the 1990s to 1.6 per 10,000 anesthetics in the 1990s–2010s; *P* = 0.83). These event rates did not differ significantly between low- and high-HDI countries either before 1990s or in the 1990s to 2010s.

Contributory anesthesia-related CA rates in high-HDI countries decreased 4.2-fold, from 2.1 per 10,000 anesthetics before the 1990s to 0.5 per 10,000 anesthetics in the 1990s to 2010s (*P* = 0.02). These event rates were not significantly different between low- and high-HDI countries in the 1990s to 2010s. Low-HDI countries were not represented before the 1990s, and analyses by country's HDI status and by high or low HDI by time period were not performed.

Perioperative CA rates decreased 1.3-fold in the high-HDI countries (8.1 per 10,000 anesthetics before the 1990s to 6.2 per 10,000 anesthetics in the 1990s–2010s; *P* < 0.001), but increased 1.2-fold those with low-HDI countries (16.4 per 10,000 before the 1990s to 19.9 per 10,000 anesthetics in the 1990s–2010s; *P* = 0.03). These event rates were 2-fold higher before the 1990s (*P* < 0.003) and 3.2-fold higher in the 1990s to 2010s (*P* < 0.001) in the low-HDI countries compared with those with high-HDI countries.

## DISCUSSION

This systematic review using meta-regression analyses showed that both anesthesia-related and perioperative CA rates decreased significantly with increasing HDI, but not with time. There was a significant reduction of totally anesthesia-related CA by time because of high-HDI country studies. In addition, the proportional meta-analyses showed a significant reduction in both anesthesia-related (3.2-fold) and perioperative (1.3-fold) CA rates in high-HDI countries, whereas the perioperative CA rates significantly increased (1.2-fold) without significant reduction in the anesthesia-related rates in those with a low HDI by time period (pre-1990s *vs* 1990s–2010s).

In contrast to a recent meta-regression analysis by time that showed a significant decline of the global perioperative CA among a total of 9,543,030 anesthetics administered to patients who underwent general anesthesia,^4^ our review, which comprised 11,975,964 patients undergoing all types of anesthesia, did not show a significant relationship between global perioperative CA and time. In contrast to the aforementioned review, our review also included many articles from the EMBASE source.

The lower anesthesia-related CA rates in high-income countries compared with low-income countries, and a greater reduction in the anesthesia-related CA rates in relation to perioperative CA rates by 2 time periods in high-income countries demonstrate great improvement in patient anesthesia safety in these countries since the early 1990s. This improvement has been attributed to a variety of safety improvements, including medications, quality of trainees, training programs, widespread adoption of practice guidelines, checklists, systematic approaches to error reduction, and specialty monitoring techniques.^8–10^ Though first developed in the early 1970s, pulse oximetry and capnography were not routinely used until a decade later in high-HDI countries.^17^ Unfortunately, even now, the routine use of pulse oximetry and capnography is not universal in some low-income countries.^1,8,18^ For high-risk patients, continued monitoring in an intensive care unit may reduce anesthesia-related morbidity and mortality; the inability to provide or failure to use these facilities may increase anesthesia-related CA and mortality rates.^19,20^

The overall rates of anesthesia-related and perioperative CA remained approximately 6.4- and 3.2-fold higher, respectively, in low-income countries compared with high-income countries in the 1990s to 2010s. These findings might have resulted from large improvements in primary care and early advancement in surgery practices in high-income countries, such as better patient selection for surgery, advances in techniques and equipment, improvements in fluid and blood management, and improved postoperative critical care.^4^ However, a study from a low-HDI country verified that many patients arrive at the OR without optimization of their disease management.^21^ According to the authors, these findings demonstrate a persistent need to improve the quantity and quality of resource utilization and access to health care, which are inadequate in low-HDI countries. Thus, intraoperative CAs and deaths that occur in patients from low-income countries might be prevented by an adequate primary care and preoperative assessment.

Certain preexisting morbidities, such as sepsis, multiple organ failure, and trauma, which occur at a higher incidence in low-income countries,^22–26^ as well as cardiovascular diseases^27^ and aging,^21^ which are more important in high-income countries, can influence the occurrence of perioperative CA. However, the lack of trained staff, essential supplies and monitoring, and low surgery rates in a context of poor basic infrastructure to support safe surgery are certainly the main factors that account for the high anesthesia-related and perioperative CA rates in low-HDI countries.^18,28^

According to Eichhorn,^9^ the challenges in patient anesthesia safety in high-income countries include preserving and extending the gains that have been obtained in improving anesthesia care and facilitating the adoption of anesthesia practice advancements in developing and underdeveloped countries. A recent review^29^ provided evidence as to the areas in which further anesthesia and patient safety-related progress can be made, such as incident reporting, standardized drug and ampoule labeling, and surgery and anesthesia checklists. The authors highlight the importance of implementing these measures in both clinical practice and medical schools to improve anesthesia safety. Nevertheless, the absence of standards does not determine that simulation-based training research has an impact on patient outcome.^30^ A study showed that poor practical application leads to critical incidents, particularly in rare events, such as intraoperative CA.^31^ Thus, continued education for anesthesia practitioners plays a pivotal role specially in rare events.

Low-HDI countries did not show a significant improvement in anesthesia-related CA rates in 1990s to 2010s *versus* pre-1990s. Differently, perioperative CA rates in low-HDI countries increased when comparing the 2 time periods. According to Ivani et al,^8^ more resources must be invested in terms of staff, equipment, recommendations, and checklists, together with a mandatory collaboration of each local government in conjunction with cooperation and assistance from high-income countries to diminish the gap that exists between the health care systems of low- and high-HDI countries.

The results of our review should be interpreted in the context of its data limitations. The different designs of the studies such as surgical populations (eg, some studies excluded pediatric patients or ASA V physical status patients), event timeframe (ie, intraoperative, first 24 postoperative hours, or 7 postoperative days), and types of surgery (some studies excluded cardiac, trauma, or obstetrical surgeries) accounted for most of the substantial heterogeneity among all analyses. Owing to the high rate of heterogeneity among studies, we used a random-effects model in the proportional meta-analysis. In addition, many reports were observational studies, whereas others used voluntary reporting registries. Some studies utilized data from a single institution, whereas others used data from nationwide surveys. Therefore, to minimize sampling bias, we included only large studies (>3000 patients), and calculated weighted event rates across all studies. Publication bias might have contributed to inadequate power to detect CA trends from the low-income countries because only a few studies before the 1990s were available from these countries. To minimize this factor, we calculated rates of change by 2 time periods within high-HDI and low-HDI country settings separately. Underreporting of perioperative and anesthesia-related cases is cited as a limitation in many studies, particularly from low-HDI countries. Indeed, if this is true, the possibility of disparity of the event rates between low- and high-HDI countries would be worse. Some relevant studies predating the searched databases or unpublished studies in an indexed journal could not be retrieved. Great disparity exists among the studies in defining anesthesia-related CA and mortality, a context that calls for the urgent development of a consensus within the specialty for standardized definitions.^32,33^

Our results showed great improvement in patient care during surgery and anesthesia in high-HDI countries but not in low-HDI countries. Subsequent reviews of anesthesia-related and perioperative CA rates should be completed periodically to provide continued global measurements of patient safety in low- and high-income countries.

In conclusion, our systematic review with meta-regression shows that both anesthesia-related and perioperative CA rates decrease with increasing HDI, but not with time. According to the proportional meta-analyses, there is a clear and consistent reduction in anesthesia-related and perioperative CA rates in high-HDI countries, but an increase in perioperative CA rates without significant alteration in the anesthesia-related CA rates in low-HDI countries by time period (pre-1990s *vs* 1990s–2010s). Therefore, anesthesia-related and perioperative CA rates remain 6.4- and 3.2-fold higher, respectively, in low-HDI countries compared with high-HDI ones in the period of the 1990s to 2010s. Global efforts and collaboration should emerge from both high- and low-income countries to improve perioperative safety and to reduce the gap that exists between their respective health care systems.
